# Seroepidemiology of human enterovirus71 and coxsackievirusA16 in Jiangsu province, China

**DOI:** 10.1186/1743-422X-9-248

**Published:** 2012-10-29

**Authors:** Hong Ji, Liang Li, YanMing Liu, HengMing Ge, XuShan Wang, JianLi Hu, Bin Wu, JianGuang Fu, ZhenYu Zhang, XiaoQin Chen, MingLei Zhang, Qiang Ding, WenBo Xu, FenYang Tang, MingHao Zhou, Hua Wang, FengCai Zhu

**Affiliations:** 1 Jiangsu Provincial Center for Disease Control and Prevention, No.172, Jiangsu Road, Gulou District, Nanjing, 210009 Jiangsu Province, China; 2 Chinese Center for Disease Control and Prevention, No.155, Changbai Road, Changping District, Beijing 102206, China; 3 Donghai County Center for Disease Control and Prevention, Jingdu Road, Niushan Town, Donghai County, Lianyungang City, Jiangsu Province, China; 4 Ganyu County Center for Disease Control and Prevention, Huanghai Road, Qingkou Town, Ganyu County, Lianyungang City, Jiangsu Province, China

**Keywords:** Human enterovirus71, CoxsackievirusA16, Maternally-acquired immunity, Neutralizing antibody, Hand, foot and mouth disease, Seroepidemiology

## Abstract

**Background:**

The major etiology of hand, foot and mouth disease (HFMD) is infection with human enterovirus A (HEV-A). Among subtypes of HEV-A, coxsackievirusA16 (CoxA16) and enterovirus 71 (EV71) are major causes for recurrent HFMD among infants and children in Jiangsu Province, mainland China. Here, we analyzed maternal antibodies between prenatal women and their neonates, to determine age-specific seroprevalence of human EV71 and CoxA16 infections in infants and children aged 0 to 15 years. The results may facilitate the development of immunization against HFMD.

**Methods:**

This study used cross-section of 40 pairs of pregnant women and neonates and 800 subjects aged 1 month to 15 years old. Micro-dose cytopathogenic effects measured neutralizing antibodies against EV71 and CoxA16. Chi-square test compared seroprevalence rates between age groups and McNemar test, paired-Samples t-test and independent-samples t-test analyzed differences of geometric mean titers.

**Results:**

A strong correlation between titers of neutralizing antibody against EV71 and CoxA16 in prenatal women and neonates was observed (r_EV71_ = 0.67, r_CoxA16_ = 0.56, respectively, *p* < 0.05). Seroprevalence rates of anti-EV71 antibody gradually decreased with age between 0 to 6 months old, remained low between 7 to 11 months (5.0–10.0%), and increased between 1 and 4 years (22.5–87.5%). Age-specific seroprevalence rates of anti-EV71 antibody stabilized in >80% of children between 5 to 15 years of age. However, seroprevalence rates of anti-CoxA16 antibody were very low (0.0–13.0%) between 0 to 6 months of age, gradually increased between 7 months to 4 years (15.0–70.0%), and stabilized at 54.0% (108/200) between 5 to 15 years. Seroprevalence rates against EV71 and CoxA16 were low under 1 year (0.0–10.0%), and showed an age dependent increase with high seroprevalence (52.5–62.5%) between 4 and10 years of age.

**Conclusions:**

Concomitant infection of EV71 and CoxA16 was common in Jiangsu Province. Therefore, development of bivalent vaccine against both EV71 and CoxA16 is critical. The optimal schedule for vaccination may be 4 to11 months of age.

## Background

Hand, foot and mouth disease (HFMD) is characterized by brief febrile episodes and characteristic skin rash, with or without oral ulcers in children. Over the last decades, many outbreaks of HFMD were reported in the Asia Pacific region, especially in areas with dense populations such as Taiwan, Japan, Malaysia, Singapore, Vietnam, Australia, South Korea, and mainland China.

In mainland China, HFMD emerged as an important public health problem, and was already classified as a category C Notifiable Infectious Disease by the Ministry of Health in China on 2 May 2008. The outbreak was mainly caused by CoxA16 and/or EV71 infection [1-3]. Compared to CoxA16, infections with EV71 appeared severe, leading to more serious complications and fatalities [4].

In humans, the major protective mechanism against EV71 and CoxA16 is cell-mediated immunity [5-8]. Humoral immunity with neutralizing antibodies is also crucial for protection against EV71 and CoxA16 infection [9-11]. Currently, immunogenicity in maternal serum and the pattern of immune responses against HFMD has not been well studied in mainland China. In addition, only a few studies on HEV71 infection have been conducted in Singapore [12], Germany [13], Vietnam [14], Taiwan [15], Brazil [16] and Japan [17]. With regards to the distribution of immunogenicity against infection with CoxA16, further study is required.

To provide fundamental data for the establishment of an immunization program against infection with EV71 and CoxA16 in China, local seroepidemiological studies are indispensable. To investigate the seroepidemiology of infection with EV71 or CoxA16 in Jiangsu province, China, we conducted a cross-sectional study. Trans-placental antibodies from prenatal women and antibodies from their neonates were collected and analyzed to identify the age-specific seroprevalence rate of natural infections with EV71 and CoxA16 in infants/children between 0 months and 15 years of age. All serum samples were collected in August 2010 from two towns in Ganyu County and two towns in Donghai County, both in LianYun Gang City, Jiang Su Province, East China.

## Results

### Titers of neutralizing antibodies to EV71 and CoxA16 by age group

**Figure 1:**
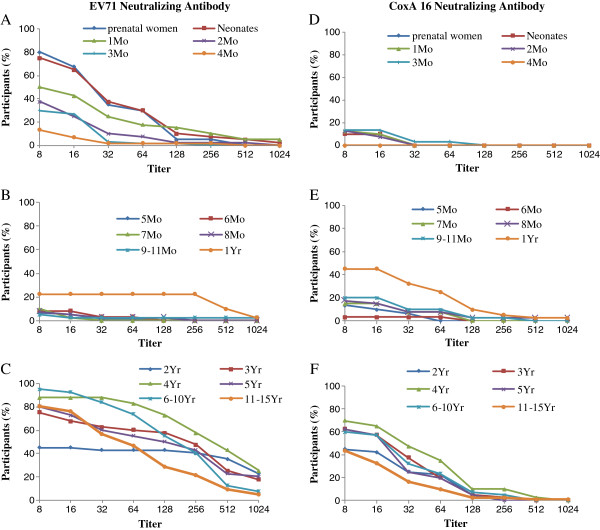
**Reverse cumulative distribution curves of neutralizing antibody titers in seropositive individuals by age group.** (**A**, **B** and **C**) The percentages of subjects with EV71 neutralizing antibody titers by age group are shown. (**D**, **E** and **F**) The percentages of subjects with CoxA16 neutralizing antibody titers by age group are shown.

The levels of neutralizing antibodies against EV71 and CoxA16 in prenatal women and their neonates were almost equally distributed. The titers against EV71 and CoxA16 decreased with age in infants aged from 1 to 9 months, while the titers increased with age in children aged from 1 to 3 years, and then reached a peak level in children at the age of 4 years. The titer level showed a decrease in children aged 5 years, 6 to 10 years and 11 to 15 years (Figure 1). In addition, from the 264 individuals with positive neutralizing antibodies against EV71 and 197 with antibodies against CoxA16, 62.3% (165/264) and 11.2% (22/197) of children aged from 1 year to 15 years demonstrated higher titers of antibody against EV71, and the titers of antibodies against CoxA16 were equal to or higher than 1:128.

### Seroprevalence rates and geometric mean titers of anti-EV71 and anti-CoxA16 in pairs of blood samples from prenatal women and neonates

The seroprevalence rates of anti-EV71 neutralizing antibodies in prenatal women and their neonates were 80.0% (32/40) and 75.0% (30/40), respectively. There was no statistically significant difference with regards to seroprevalence rates of anti-EV71 antibodies between prenatal women and their neonates (*p* = 0.688, by McNemar test). The geometric mean titers (GMT) of anti-EV71 antibodies in prenatal women and their neonates were 18.7 (95%CI: 12.9-27.1) and 20.0 (95%CI: 12.8-31.4), respectively. Again, there was no statistically significant difference between the two means (Paired-samples test = 0.408, df = 39; *p* = 0.685). In addition, there was a correlation between anti-EV71 antibody titers in neonates and anti-EV7 antibody titers in their mothers (r = 0.67, *p* < 0.01).

The seroprevalence rates of anti-CoxA16 neutralizing antibodies in these prenatal women and their neonates were 12.5% (5/40) and 10.0% (4/40), respectively. No statistically significant difference in seroprevalence rates of anti-CoxA16 antibodies was observed between these two groups (*p* = 1.000, by McNemar test). Furthermore, the GMTs of anti-CoxA16 antibodies were 4.5 (95%CI: 4.0-5.1) in prenatal women and 4.6 (95%CI: 4.0-5.3) in their neonates. There was no statistically significant difference between these two mean values (Paired-samples test = 0.298; df = 39; *p* = 0.767). The titers of anti-CoxA16 antibody in neonates correlated with the titers of anti-CoxA16 antibodies in their mothers (r = 0.56, *p* < 0.01), (Table 1).

**Table 1:** Relationship of anti-EV71/anti-CoxA16 antibodies in pairs of blood samples collected from prenatal women and their neonates

**Neutralizing antibody titers of neonates**	**Neutralizing antibody titers of prenatal women**		**Total titers**
**anti-EV71**			
	<1:8	≥1:8	
<1:8	6(15.0)	4(10.0)	10(25.0)
≥1:8	2(5.0)	28(70.0)	30(75.0)
Total	8(20.0)	32(80.0)	40(100.0)
**anti-CoxA16**			
<1:8	33(82.5)	3(7.5)	36(90.0)
≥1:8	2(5.0)	2(5.0)	4(10.0)
Total	35(87.5)	5(12.5)	40(100.0)

### Seroprevalence rates and GMTs of anti-EV71/anti-CoxA16 by age group

**Figure 2:**
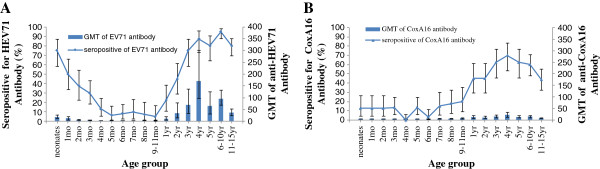
**Seroprevalence rates of neutralizing antibodies and geometric mean titers (GMT) by age group.** (**A**) Seroprevalence rates of neutralizing antibodies and GMT of EV71 by age group. (**B**) Seroprevalence rates of neutralizing antibodies and GMT of CoxA16 by age group.

The seroprevalence rates of anti-EV71 antibodies showed a gradual decrease in infants aged 0 to 6 months old, ranging from 75.0% to 6.7%. There was a significant drop in seroprevalence rates in infants aged 0 to 6 months old (*p* = 0.000). The seroprevalence rates were relatively low (5.0–10.0%) in infants aged from 7 month to 9–11 months old. No statistically significant difference in seroprevalence rate was shown in this age group (*p* = 0.697). The seroprevalence rates showed a gradual rise from 22.5% in children aged 1 year old to 87.5% in children aged 4 years old, and thereafter reached a plateau in over 80.0% of children older than five years of age (Figure 2A).

The seroprevalence rate of anti-CoxA16 antibody was low in infants younger than 6 months of age (0.0–13.3%), compared with those of anti-EV71 antibodies. The mean seroprevalence rate was 17.5% in infants aged from 7 to 11 months old. There were no statistically significant differences in seroprevalence rates either in infants aged 0 to 6 months old (*p* = 0.053) or in infants aged 7 to 11 months old (*p* = 0.841). The seroprevalence rates of anti-CoxA16 antibodies gradually increased from 45.0% in children aged 1 year old to 70.0% in children aged 4 years old, and then was greater than 60.0% in children aged 5 years and 6 to 10 years old. It declined to 43.8% in children aged 11 to 15 years old. Together, the seroprevalence rate of anti-CoxA16 antibody demonstrated a similar pattern to anti-EV71 antibodies (Figure 2B). The GMTs of anti-EV71 antibodies decreased from neonates to infants at the age of 6 months (from 20.0 to 4.4), and then remained at a low but relatively steady state in infants aged 7 to 11 months old. The GMTs of anti-CoxA16 antibody were lower than for anti-EV71 antibodies and maintained a relatively stable level in neonates to infants aged 9 to 11 months old. The GMTs of anti-EV71 and anti-CoxA16 antibodies both gradually increased in children aged from 1 year to 4 years old, reached their peak levels in children aged 4 years old, and then declined (Figure 2).

If we consider the individuals aged 0 to 6 months old, 7 to 11 months old, 1 to 3 years old and 4 to 5 years old altogether, the pooled seroprevalence rates of anti-EV71 and anti-CoxA16 antibodies were 34.3% (233/680, 95%CI:30.7-38.0) and 24.6% (167/680, 95%CI:21.4-28.0), respectively. The seroprevalence rates and GMTs of anti-EV71 and anti-CoxA16 antibodies were relatively low in infants aged 0 to 11 months old compared with the other two age groups. The seroprevalence rates of anti-EV71 and anti-CoxA16 antibodies in children aged 4 to 5 years old were both significantly higher than those observed in children aged 1 to 3 years old (*p <* 0.05). The GMTs of anti-EV71 and anti-CoxA16 antibodies in children aged 4 to 5 years old were higher than those in children aged 1 to 3 years old, but the GMTs of anti-CoxA16 antibodies showed no statistically significant difference between children aged 1 to 3 years old and children aged 4 to 5 years old (*p* > 0.05) (Table 2).

**Table 2:** Seroprevalence rates and GMTs of EV71 and CoxA16 neutralizing antibodies among age groups

**Neutralizing antibody**	**0–6-month-old**	**7–11-month-old**	**1–3-year-old**	**4–5-year-old**
**EV71**	**Seroprevalence rates (95%CI)**			
	27.8	7.5	47.5	83.8
	(23.2-37.8)	(3.5-13.8)	(38.3-56.8)	(73.9-91.1)
	**GMTs (95%CI)**			
	6.8	4.5	30.2	105.8
	(6.1-7.6)	(4.1-5.1)	(19.8-46.0)	(68.2-164.2)
**CoxA16**	**Seroprevalence rates (95%CI)**			
	8.9	17.5	50.8	66.3
	(6.2-12.3)	(11.2-25.5)	(41.6-60.0)	(54.8-76.4)
	**GMTs (95%CI)**			
	4.6	5.9	12.6	16.6
	(4.4-4.8)	(5.0-7.0)	(9.9-15.9)	(12.4-22.1)

### Age-dependent immunity to HFMD

**Figure 3:**
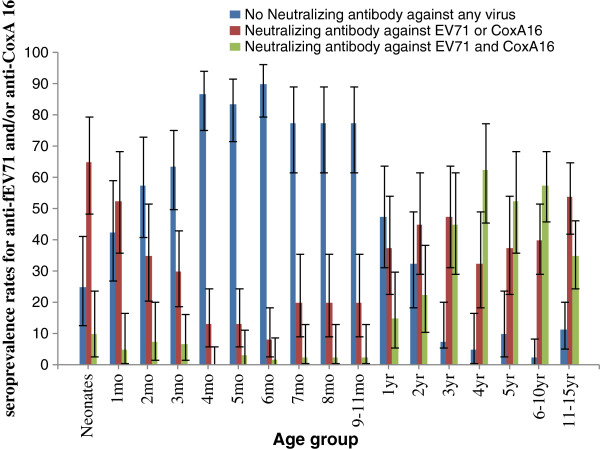
Age-specific fractions of individuals revealing complete, partial, or no immunity to EV71/CoxA16 infections.

The seroprevalence rates of neutralizing antibodies against both EV71 and CoxA16 were relatively low in neonates to infants aged 9–11 months old (0.0–10.0%), and gradually increased in children aged from 1 years old to 4 years old. Immunity against EV71 and CoxA16 was relatively higher in children aged 4 to 10 years old (52.5–62.5%), nonetheless, it declined to 35.0% in children aged 11 to 15 years old. The seroprevalence rates against either EV71 or CoxA16 decreased gradually in neonates and infants at the age of 6 months (from 65.0% to 8.3%), and the individual (either EV71 or CoxA16) age-specific seroprevalence rates became steady in more than 30.0% of children aged 1 to 15 years old. The seroprevalence rates against neither EV71 nor CoxA16 increased in neonates to infants aged 1–6 months old, but reached a peak value (90.0%) in infants at the age of 6 months, and then gradually declined with age, the rates ranged between 32.5% and 77.5% in children aged from 7 months to 2 years old, and then decreased to 2.5% and 11.3% in children aged from 3 years to 15 years old (Figure 3).

## Discussion

This study describes maternally-derived and age-specific seroprevalence of anti-EV71 and anti-CoxA16 neutralizing antibodies in infants/children aged from neonates to 15 years old. Retrospective seroepidemiology indicated a geographical difference in EV71 and CoxA16 infection [18]. The results of this study revealed that EV71 infections occurred more frequently than CoxA16 infections in children over 2 years of age in China.

In mice, trans-placental transfer of maternal antibodies following maternal immunization against EV71 protected newborn mice against lethal infection [19]. In humans, the presence of maternal anti-EV71 antibodies was also demonstrated in neonates, and the seroprevalence rates and antibody titers of both antibodies demonstrated some correlation with those in their mothers [15]. Our data also suggest a similar correlation with maternal anti-EV71 antibodies. Unfortunately, the data obtained from studies focused on anti-CoxA16 seemed to be very rare. A cohort study in mainland China revealed a pattern of age-related decline of anti-CoxA16 antibody levels, similar to that of anti-EV71 antibody levels [20]. In addition, the titers and GMTs against EV71 and CoxA16 were almost equally distributed. It has been suggested that if mothers show higher levels of anti-EV71 and/or anti-CoxA16 antibodies, the antibody titers transferred vertically from mothers to their infants are also higher.

Previous reports showed that 23.7% of HFMD patients showed anti-EV71 antibody titers ≥1:128, whereas none of the healthy children showed such titers [8]. A recent prospective cohort study showed that 29% of EV71 infections were asymptomatic [21]. To our surprise, our study found that 62.3% of children aged over 1 year old later developed anti-EV71 antibody titers ≥1:128, although none of these children were confirmed with a clinical diagnosis of HFMD at a hospital or health care provider. This implies that clinically silent or almost silent disease is the most common manifestation of EV71 infection in infants and children in China.

Our data shows that only 11.2% of children aged more than 1 year old had anti-CoxA16 antibody titers ≥1:128. This observation reflects the absence of exposure to CoxA16. A lower seroprevalence rate of anti-CoxA16 compared to anti-EV71 antibodies in children aged 2 to 15 years is also supportive of our previous observation.

This study and several seroepidemiological studies in humans have revealed that the seroprevalence rates of anti-EV71 antibodies increased with age [11,22,23]. Our study not only provided data supportive of age-related seroprevalence of EV71 infection, but also suggested a similar age-related seroprevalence of CoxA16 infection. The seroprevalence rates of EV71 and/or CoxA16 were relatively low in children aged 4 to 11 months of age. In 70.2% (337/480) infants aged from neonate to 9 to 11 months of age there were no antibodies against either EV71 or CoxA16; thus, the high incidence of infection in younger age groups is attributable to the lack of protective antibodies [12,23-26]. A cross-sectional study in Singapore also indicated that the infection was mostly acquired in pre-school years, with an annual infection rate of 12% [10]. In our study, most children aged 2 to 5 years old received pre-school education in kindergartens, and demonstrated higher seroprevalence rates of EV71 and CoxA16, and this finding was supported by the highest incidence of HFMD in preschool years below 5 years of age [27]. Hence, strengthening health improvement measures in kindergartens is required to effectively control infection. Furthermore, the GMTs and neutralizing titers reached peak levels in children aged 4 years old, and thereafter gradually declined with age, but were still relatively high, indicating reinfection with the same strain was rare and that neutralizing antibody titers were higher at early stages of natural infection. This is consistent with previous studies [8,13]. Our data also demonstrated that the population immunity against EV71 and CoxA16 was relatively high in children aged 4 to 10 years old (52.5–62.5%), suggesting that EV71 and CoxA16 infections are highly prevalent in this age group.

Based on historical experiences with poliovirus vaccines and several EV71 vaccine candidates that were evaluated in animals [28], several phase I clinical trials to test EV71 vaccine candidates were conducted in Singapore [29], Taiwan [30] and mainland China [31-36]. Some Phase II clinical trials for EV71 vaccine candidates have also been conducted in mainland China [37] between 2010–2011. The inactivated whole-virus vaccine candidates may be available for clinical use in the next 5 to 10 years [28].

## Conclusion

In conclusion, our data demonstrated that the optimal time for vaccination against EV71 should be infantile periods from month 4 to 11. However, if we take into account the higher co-infection rates in mainland China and the probably weak cross-protection of immunity to EV71 with CoxA16 [38], the development of a bivalent vaccine which provides protection against both EV71 and CoxA16 antigens should provide a more global coverage for HFMD.

## Methods and materials

### Human subjects and serum samples

The collected serum samples were analyzed to measure the titers of neutralizing antibody against EV71 and CoxA16 in prenatal women and their neonates at birth (cord blood). A total of 40 pairs of serum samples were collected. To further explore the age-related seroprevalence of neutralizing antibodies against EV71 and CoxA16 in infants and young children, 16 more age groups were selected: 1, 2, 3, 4, 5, 6,7, 8 and 9 to 11 months, as well as 1, 2, 3, 4, 5, 6 to 10 and 11 to 15 years of age, respectively. Serum samples from 400 infants and children at the age of 1, 2, 7, 8, 9 to 11 months and 1, 2, 3, 4, 5 years were collected, with 40 individuals in each of the abovementioned age group. Samples from 240 infants from 3, 4, 5 and 6 months of age were collected, with 60 infants in each of the abovementioned age groups. Samples from 160 children at the age of 6 to 10 and 11 to 15 years were also collected, with 80 children in each of the abovementioned age groups. All 800 serum samples were collected randomly in Ganyu County and Donghai County in August 2010.

Medical history and related clinical data were collected through interviews with the parents/guardians of each infant or child before samples were collected. No subject showed symptoms or signs of HFMD at the time of sample collection. Written informed consent was obtained from each participant or from his or her parent/guardian. The study protocol was approved by the ethics committee of the Jiangsu Provincial Center for Disease Control and Prevention, and the study was conducted in compliance with the principles of the Declaration of Helsinki.

### Measurement of EV71 and CoxA16 Neutralizing Antibodies

In this study, EV71/Fuyang/m01/2008 (C4 genotype) and CoxA16 /G-10 (A genotype) were used to quantify neutralizing antibody levels of anti-EV71 and anti-CoxA16 by micro-neutralization assay. Blood samples were diluted to a ratio of 1:8, and then were inactivated at the temperature of 56°C for 30 minutes. The serum was serially diluted from 1:8 to 1:2048, and was thereafter mixed with 100 TCID_50_ of EV71 and CoxA16 equally. Then serum was added into a 96-micro plate at 37°C for 2 hours, and finally an RD cell suspension (1 × 10^5^ cells/ml) was added. Cell control, serum control, virus control and virus back titration (if the result of backdrop is 32 ~ 320 TCID_50_/well, the test is successful) were included in each plate and incubated in a CO_2_ incubator at 35°C for 7 days. Cytopathogenicity was observed by microscopy to identify titers of neutralizing antibodies, defined as the inhibition of 50% cytopathogenic effect. This method was previously calibrated using reference sera of EV71 and CoxA16, and no cross-reaction was found. Neutralizing antibodies against EV71 and CoxA16 were considered positive, if the dilution was equal to or greater than 1:8 [15,22,39].

### Statistical analysis

The titers of neutralization antibodies were log-transformed to calculate the GMTs with 95% confidence intervals. The statistical association between these two variables was tested by McNemar test. Paired-samples t-test and Independent-samples t-test were utilized to analyze the difference of GMTs. Chi-square test was used to compare the seroprevalence rates between age groups. All titers below 1:8 were assumed to be 1:4 for calculation. The titers beyond 1:2048 were designated 1:2048. Hypothesis testing was conducted by two-sided tests, with an alpha value set at 0.05 to define statistical significance. All statistical analyses were performed via Microsoft Excel 2007 (Microsoft, Redmond, WA, USA) or Stata version 10.0 (StataCorp, College Station, TX).

## Abbreviations

EV71: Enterovirus 71; CoxA16: CoxsackievirusA16; HFMD: Hand, foot and mouth disease; GMT: Geometric mean titer.

## Competing interests

All authors declare that they have no competing of interests.

## Authors’ contributors

HJ prepared manuscript, FCZ, MHZ, HW and YML designed the study and organized coordination, FYT, LL and JLH performed the field study, HMG, XSW, ZYZ, XQC and MLZ contributed to subject recruitment, WBX, BW,GJF and QD prepared and performed the experiments. All authors contributed to the collection and analysis of the data and to the preparation of the report. All authors read and approved the final manuscript.

## References

[B1] Ooi MH Wong SC Podin Y Akin W del Sel S Mohan A Chieng CH Perera D Clear D Wong D Blake E Cardosa J Solomon T Human Enterovirus 71 Disease in Sarawak, Malaysia: A Prospective Clinical, Virological, and Molecular Epidemiological Study Clin Infect Dis 2007 44 646 656 10.1086/511073 17278054

[B2] Bible JM Pantelidis P Chan PK Tong CY Genetic evolution of enterovirus 71: epidemiological and pathological implications Rev Med Virol 2007 17 371 379 10.1002/rmv.538 17487831

[B3] Yan JJ Su IJ Chen PF Liu CC Yu CK Wang JR Complete Genome Analysis of Enterovirus 71 Isolated from an Outbreak in Taiwan and Rapid Identification of Enterovirus 71and Coxsackievirus a16 by RT-PCR J Med Virol 2001 65 331 339 10.1002/jmv.2038 11536241

[B4] Chang LY Lin TY Huang YC Tsao KC Shih SR Kuo ML Ning HC Chung PW Kang CM Comparison of enterovirus71, coxsackie-virusA16 clinical illnesses during the Taiwan enterovirus epidemic Pediatr Infect Dis J 1999 18 1092 1096 10.1097/00006454-199912000-00013 10608631

[B5] Yang KD Yang MY Li CC Lin SF Chong MC Wang CL Chen RF Lin TY Altered cellular but not humoral reactions in children with complicated enterovirus 71 infections in Taiwan J Infect Dis 2001 183 850 856 10.1086/319255 11237800

[B6] Chang LY Hsiung CA Lu CY Lin TY Huang FY Lai YH Chiang YP Chiang BL Lee CY Huang LM Status of cellular rather than humoral immunity is correlated with clinical outcome of enterovirus 71 Pediatr Res 2006 60 466 471 10.1203/01.pdr.0000238247.86041.19 16940249 PMC7086547

[B7] Juhela S Hyöty H Lönnrot M Roivainen M Simell O Ilonen J Enterovirus infections and enterovirus specific T-cell responses in infancy J Med Virol 1998 54 226 232 10.1002/(SICI)1096-9071(199803)54:3<226::AID-JMV14>3.0.CO;2-F 9515773

[B8] Yang C Deng C Wan J Zhu L Leng Q Neutralizing antibody response in the patients with hand, foot and mouth disease to enterovirus 71 and its clinical implications Virol J 2011 8 306 10.1186/1743-422X-8-306 21679417 PMC3142239

[B9] Yu CK Chen CC Chen CL Wang JR Liu CC Yan JJ Su IJ Neutralizing antibody provided protection against enterovirus type 71 lethal challenge in neonatal mice J Biomed Sci 2000 7 523 528 10.1007/BF02253368 11060501

[B10] Foo DG Alonso S Chow VT Poh CL Passive protection against lethal enterovirus 71 infection in newborn mice by neutralizing antibodies elicited by a synthetic peptide Microbes Infect 2007 9 1299 1306 10.1016/j.micinf.2007.06.002 17890123

[B11] Wu TC Wang YF Lee YP Wang JR Liu CC Wang SM Lei HY Su IJ Yu CK Immunity to avirulent enterovirus 71 and coxsackie A16 virus protects against enterovirus 71 infection in mice J Virol 2007 81 10310 10315 10.1128/JVI.00372-07 17626076 PMC2045469

[B12] Ooi EE Phoon MC Ishak B Chan SH Seroepidemiology of Human Enterovirus 71, Singapore Emerg Infect Dis 2002 8 995 997 10.3201/eid0809.010397 12194783 PMC2732542

[B13] Rabenau HF Richter M Doerr HW Hand, foot and mouth disease: seroprevalence of Coxsackie A16 and Enterovirus 71 in Germany Med Microbiol Immunol 2010 199 45 51 10.1007/s00430-009-0133-6 19941005

[B14] Tran CB Nguyen HT Phan HT Tran NV Wills B Farrar J Santangelo JD Simmons CP The seroprevalence and seroincidence of enterovirus71 infection in infants and children in Ho Chi Minh City Viet Nam. PLoS One 2011 6 e21116 10.1371/journal.pone.0021116 PMC3134465 21765891

[B15] Luo ST Chiang PS Chao AS Liou GY Lin R Lin TY Lee MS Enterovirus 71 maternal antibodies in infants, Taiwan Emerg Infect Dis 2009 15 581 584 19331737 10.3201/1504.081550 PMC2671432

[B16] Gomes Mde L de Castro CM Oliveira MJ da Silva EE Neutralizing antibodies to enterovirus 71 in Belem, Brazil Mem Inst Oswaldo Cruz 2002 97 47 49 11992146 10.1590/s0074-02762002000100006

[B17] Hagiwara A Tagaya I Komatsu T Seroepidemiology of enterovirus 71 among healthy children near Tokyo Microbiol Immunol 1979 23 121 124 223018 10.1111/j.1348-0421.1979.tb00448.x

[B18] Zhu Z Zhu S Guo X Wang J Wang D Yan D Tan X Tang L Zhu H Yang Z Jiang X Ji Y Zhang Y Xu W Retrospective seroepidemiology indicated that human enterovirus 71 and coxsackievirus A16 circulated wildly in central and southern China before large-scale outbreaks from 2008 Virol J 2010 7 300 10.1186/1743-422X-7-300 21050463 PMC2989968

[B19] Chiu CH Chu C He CC Lin TY Protection of neonatal mice from lethal enterovirus 71 infection by maternal immunization with attenuated Salmonella enterica serovar Typhimurium expressing VP1 of enterovirus 71 Microbes Infect 2006 8 1671 1678 10.1016/j.micinf.2006.01.021 16815726

[B20] Mao QY Liao XY Yu X Li N Zhu FC Zeng Y Liang ZL Li FX Wang JZ Lu FM Zhuang H Dynamic change of mother-source neutralizing antibodies against enterovirus 71 and coxsackievirus A16 in infants Chin Med J (Engl) 2010 123 1679 1684 20819628

[B21] Lee MS Chiang PS Luo ST Huang ML Liou GY Tsao KC Lin TY Incidence rates of enterovirus 71 infections in young children during a nationwide epidemic in Taiwan, 2008–09 PLoS Negl Trop Dis 2012 6 e1476 10.1371/journal.pntd.0001476 22348156 PMC3279337

[B22] Chang LY King CC Hsu KH Ning HC Tsao KC Li CC Huang YC Shih SR Chiou ST Chen PY Chang HJ Lin TY Risk factors of enterovirus 71 infection and associated hand, foot, and mouth disease/herpangina in children during an epidemic in Taiwan Pediatrics 2002 109 e88 10.1542/peds.109.6.e88 12042582

[B23] Diedrich S Weinbrecht A Schreier E Seroprevalence and molecular epidemiology of enterovirus 71 in Germany Arch Virol 2009 154 1139 1142 10.1007/s00705-009-0413-x 19506798

[B24] Castro CM Cruz AC Silva EE Gomes Mde L Molecular and seroepidemiologic studies of enterovirus 71 infection in the State of Para, Brazil Rev Inst Med Trop São Paulo 2005 47 65 71 10.1590/s0036-46652005000200002 15880216

[B25] Ho M Enterovirus 71: the virus, its infections and outbreaks J Microbiol Immunol Infect 2000 33 205 216 11269363

[B26] Lu CY Lee CY Kao CL Shao WY Lee PI Twu SJ Yeh CC Lin SC Shih WY Wu SI Huang LM Incidence and case-fatality rates resulting from the 1998 enterovirus 71 outbreak in Taiwan J Med Virol 2002 67 217 223 10.1002/jmv.2210 11992582

[B27] Ang LW Koh BK Chan KP Chua LT James L Goh KT Epidemiology and control of hand, foot and mouth disease in Singapore, 2001–2007 Ann Acad Med Singapore 2009 38 106 112 19271036

[B28] Lee MS Chang LY Development of enterovirus 71 vaccines Expert Rev Vaccines 2010 9 149 156 10.1586/erv.09.152 20109026

[B29] Safety and Immunogenicity Study of an Inactivated Vaccine Against Hand, Foot and Mouth Disease Caused by Enterovirus 71 http://clinicaltrials.gov/ct2/show/NCT01376479

[B30] A Study to Evaluate the Safety and Immunogenicity of EV71 Vaccine http://clinicaltrials.gov/ct2/show/NCT01268787

[B31] A Safety Study of Inactivated EV71 Vaccine (Human Diploid Cell, KMB-17) in Chinese Adults, Children and Infants http://clinicaltrials.gov/ct2/show/NCT01391494

[B32] A Clinical Trial for Inactivated Vaccine (Vero Cell) Against EV71 in Chinese Children and Infants http://clinicaltrials.gov/ct2/show/NCT01313715

[B33] Safety of an Inactivated Enterovirus Type 71 Vaccines in Healthy Children http://clinicaltrials.gov/ct2/show/NCT01273246

[B34] Safety of an Inactivated Enterovirus Type 71 Vaccine in Healthy Adults http://clinicaltrials.gov/ct2/show/NCT01273233

[B35] A Clinical Trial for Inactivated Vaccine (Vero Cell) Against EV71 in Chinese Healthy Young Adults and Children http://clinicaltrials.gov/ct2/show/NCT01267903

[B36] Safety and Immunogenicity of an Inactivated EV71 Vaccine in Infants http://clinicaltrials.gov/ct2/show/NCT01421121

[B37] A Phase II Clinical Trial for Inactivated Vaccine (Vero Cell) Against EV71 in Chinese Children and Infants http://clinicaltrials.gov/ct2/show/NCT01399853

[B38] Huang WC Huang LM Kao CL Lu CY Shao PL Cheng AL Fan TY Chi H Chang LY Seroprevalence of enterovirus 71 and no evidence of crossprotection of enterovirus 71 antibody against the other enteroviruses in kindergarten children in Taipei city J Microbiol Immunol Infect 2011 in press 10.1016/j.jmii.2011.09.025 22154997

[B39] Hsu CH Lu CY Shao PL Lee PI Kao CL Chung MY Chang LY Huang LM Epidemiologic and clinical features of non-polio enteroviral infections in northern Taiwan in 2008 J Microbiol Immunol Infect 2011 44 265 273 10.1016/j.jmii.2011.01.029 21524954

